# Cardioprotection during Adult and Pediatric Open Heart Surgery

**DOI:** 10.1155/2015/712721

**Published:** 2015-06-23

**Authors:** M-Saadeh Suleiman, Malcolm Underwood, Hajime Imura, Massimo Caputo

**Affiliations:** ^1^Bristol Heart Institute, Level 7, Bristol Royal Infirmary, Faculty of Medicine & Dentistry, University of Bristol, Bristol, UK; ^2^Cardiothoracic Surgery, The Chinese University of Hong Kong, Faculty of Medicine, Prince of Wales Hospital, Shatin, Hong Kong; ^3^Cardiovascular Surgery, Nippon Medical School, 1-1-5 Sendagi Bunkyo-ku, Tokyo 113-8603, Japan; ^4^Congenital Heart Surgery, Royal Hospital for Children and University of Bristol, Level 7, Bristol Royal Infirmary, Marlborough Street, Bristol BS2 8HW, UK

Myocardial reperfusion damage following cardioplegic ischemic arrest is a key determinant of postoperative organ functional recovery, morbidity, and mortality in adult and pediatric patients undergoing open heart surgery. The vulnerability of the diseased heart to ischemia and reperfusion is different for different pathologies or associated disease (e.g., coronary disease, hypertrophy, diabetes, etc.) and different age (e.g., neonate, infant, children, and adult). These differences and the changing nature of adult patients (e.g., aging population) present a major challenge in translating novel interventions. Thus far, hyperkalemic cardioplegic solutions, which by arresting the heart preserve substrates and delay the onset of the ischaemic insult, remain the corner stone for cardioprotection during open heart surgery. Ongoing strategies to improve myocardial protection include the inclusion of various additives that aim at reducing the damaging effects of ischemia and reperfusion (e.g., calcium overload, metabolic derangement, and accumulation of reactive oxygen species). Recent and novel strategies have also included gene and cell therapies.

In this special issue, several reviews and research articles have provided novel interpretations and data to help in the search for designing an optimal strategy to reduce myocardial injury during cardiac surgery and thus improve long term cardiac functional recovery. For example, a strong argument has been made for the potential role of inhibiting monoamine oxidases (MAOs) in cardioprotective strategies (O. M. Duicu et al.). The activity of this enzyme is linked to oxidative stress and the central role of mitochondria in disease and death. It is therefore recommended to test this in a prospective study in cardiac patients with and without diabetes undergoing heart surgery. In their review, A. Habertheuer et al. point out the importance of the changing characteristics of cardiac surgery patients and propose that better understanding of the associated molecular changes could offer new directions for the design of new more appropriate cardioprotective regimens.

N. Lakusic et al. address the very interesting topic linking changes in heart rate variability after coronary artery bypass grafting to postoperative morbidity. There is clearly an important role of the autonomic nervous system in the consequences of ischemia/reperfusion injury. They emphasize the fact that several studies have shown a reduction in heart rate variability after coronary artery bypass grafting surgery. They point out the need for a study investigating the link between decreased heart rate variability and the outcome of coronary artery bypass graft surgery patients. R. Wagner et al. focus on myocardial conditioning and its therapeutic cardioprotective potential. In this respect, they point out that despite the extensive experimental studies, almost all cardioprotective therapies have failed in the third phase of clinical trials. They propose that the evolutionary young cellular mechanisms of protection against oxygen handling are not very robust. An experimental study by T. Sato et al. suggests that insulin activated survival pathways facilitate preservation of cardiac contractility during ischemia-reperfusion injury in the isolated rat heart in a way that could be similar to conditioning-induced protection.

E. W. Kuhn et al. present data from a pilot trial investigating the effect of cardioplegia temperature on endothelial injury in patients undergoing on-pump coronary artery bypass graft surgery. They demonstrate perioperative endothelial injury and showed that cold is better than warm blood cardioplegia. A relevant review by Q. Yang et al. points out that coronary endothelial dysfunction occurring during cardiac surgery could be due to functional alteration of endothelial channels and that these channels could be potential targets for endothelial protection during cardiac surgery.

The developing heart and myocardial protection during pediatric cardiac surgery is an area in need of more research. A. Mokhtari and M. Lewis address the very important issue of controlled reoxygenation in cyanotic paediatric patients undergoing open heart surgery. The finding that cardiopulmonary bypass triggers cardiac injury prior to cardioplegic arrest [1] highlights the need for controlling reoxygenation during cardiopulmonary bypass. Recent studies have successfully demonstrated the benefits of this approach [2]. M. Cherif et al. investigated the involvement of Gab1 (Grb2 associated binding protein 1), a protein required for fibroblast cell survival and in maintaining cardiac function. They showed that this protein was upregulated in hearts of cyanotic children possibly as part of survival signaling response to hypoxia. Finally, M. Shirakawa et al. have provided evidence showing that propofol at a clinically relevant concentration is cardioprotective in the immature heart. This anesthetic has already been shown to be protective in adult models when used in cardioplegia [3] and has been included in cardioplegic solutions during surgery in patients with isolated coronary artery bypass grafting or aortic valve replacement using cardiopulmonary bypass [4].

We hope that this special issue provides the readers with new insights into different approaches used to protect the adult and the pediatric heart against the damaging effects of ischemic and reperfusion injury. If anything, the work described emphasizes the need for a more comprehensive strategy taking into account pathologies and age.

*M-Saadeh Suleiman* *Malcolm Underwood* *Hajime Imura* *Massimo Caputo*
